# Metabolic set theory: a generalized model of microbial interactions

**DOI:** 10.1038/s41540-026-00774-4

**Published:** 2026-07-01

**Authors:** Jyoti Jyoti, Hannah Zoller, Wolfgang zu Castell, Marc-Thorsten Hütt

**Affiliations:** 1 School of Science Constructor University Bremen, Bremen, Germany; 2 SRC Stockholm Resilience Centre, Stockholm, Sweden; 3 Department Geoinformation, GFZ Helmholtz Centre for Geosciences, Telegrafenberg, Potsdam, Germany; 4 Department of Statistics, Ludwig-Maximilians-University Munich, München, Germany

**Keywords:** Computational biology and bioinformatics, Mathematics and computing, Microbiology, Systems biology

## Abstract

Understanding the composition of microbial communities in their environment remains a challenge due to the complex interplay of factors like inter-species interactions and nutrient availability. In this context, it has become an established approach to use overlap in functional subsets of metabolic networks as indices of synergy and competition among microorganisms. Here, we show that this idea can actually be reduced to a much simpler principle. Leveraging the agent-based community modeling software BacArena and natural co-occurrence patterns in the human gut microbiome for a systematic comparison, we find that simple set-theoretical indices explain interactions to a similarly high degree as more sophisticated, established approaches based on network topology. Furthermore, we observe that the performance of most indices decreases substantially for patients diagnosed with obesity or inflammatory bowel disease, suggesting a systemic decline in the microbiome.

## Introduction

Metabolism shapes the dynamics of microbial ecosystems^1^. The intricate interplay of metabolic competition and facilitation lies at the heart of ubiquitous compositional patterns in natural microbial communities^2,3^. With its relevance in health and disease, one example of a microbial ecosystem, the human microbiome, has become an object of intense research in Systems Biology and at the interface of Systems Biology and Ecology^4^. Still, we do not yet have a ‘theory of the microbiome’, but rather a wide range of concepts and tools to statistically infer microbial interaction networks from abundance patterns^5–8^. Although it is still controversial to what extent co-occurrence data is imprinted by interactions between species^9^, traces of interactions could be identified in co-occurrence data in both in-silico and in-vivo experiments^10,11^.

On a more structural level, it has been shown that information on metabolic strategy and ecosystem function of an organism can be identified from the topology of metabolic networks^12–14^. In spite of this progress, the diversity of microbial interactions and the dynamic interplay between host and microbiome^15^ still leave us far from a complete understanding of explicit metabolic interactions and their role in explaining the composition, stability, and ecosystem functioning of microbial communities^16^.

An influential study conducted by the Borenstein lab^17,18^ is considered a key step towards a mechanistic understanding of synergies and competition among microorganisms, showing that simple principles based on the organisms’ metabolic systems govern the main features of microbial interactions. Following a heuristic approach, the authors identified key indicators of competition and synergy between two species by categorizing metabolites into the two levels of a *seed set* and a complementary *product set* based on their distinct topological properties. Comparing the overlap of the same and different levels with natural co-occurrences demonstrates the predictive capability of these indices and illustrates their potential to distinguish interaction-driven from habitat-filtered communities.

In this study, we show that this idea can actually be reduced to a much simpler principle. Using the hierarchical organization of metabolic systems^19,20^ to define sets of metabolites – or even the mere distinction between the outside and inside of a cell – we claim that competition is based on the overlap of sets from the *same* level, while synergy is determined by the overlap of sets from *different* levels. To measure the degree of overlap for pairs of metabolic networks, we introduce scoring functions, hereafter called *indices*. We study a range of implementations of this basic principle and functionally validate the resulting indices using the microbial simulation platform BacArena^21^, as well as co-occurrence patterns of microorganisms in the human microbiome. Our choice of BacArena as a simulation tool combining FBA with agent-based modeling is based on the proven value of these instruments in bridging the gap between individual metabolic strategies and ecosystem dynamics^22,23^. In particular, this choice allows us to explore metabolic activity without further assumptions on abundance ratios or community objectives, which are typically required in classical community FBA approaches^24^. In this sense, we consider BacArena as closer to reality than classical compartmentalized models.

Similar to classical approaches in macroecology, our *Metabolic set theory* provides an important tool to capture complex metabolic understanding within easy-to-compute, higher-level aggregates, which can then be utilized in subsequent ecological analysis. Furthermore, our approach demonstrates how metabolic hypotheses can be built into appropriate indices to stratify host phenotypes in microbiome-induced diseases.

## Results

At the core of our study are the interaction indices, which we derive from the genome-based metabolic networks (GEMs) of 73 prevalent gut microbial species. The metabolic networks are reconstructed using GEMs from the Virtual Metabolic Human (VMH) database (vmh.life)^25^. For each species, reactions were extracted from the model and used to construct a directed unweighted network, where an edge (*m*_1_, *m*_2_) exists if *m*_1_ is a reactant and *m*_2_ is a product in at least one reaction (see *Methods* for details).

In the following, we want to answer two questions: Do the indices indeed capture competition and synergy? And can the indices serve as indicators of community composition? We approach these questions from two directions. In an in-silico study, we simulate co-cultures of the microbial species using the agent-based modeling framework BacArena^21^. This set-up enables a neat isolation of pairwise effects and, therefore a suitable first testing environment. In order to avoid spatial and temporal constraints, we measure competition and synergy via pairwise patterns of shared feeding and cross-feeding, respectively, rather than via a more classical biomass-based approach. Feeding patterns are averaged across simulations varying in richness and composition of the initial medium. Minimal media, which ensure non-zero growth, are derived from flux balance analysis. We quantify the relationship between metabolic activity and pairwise indices via correlation analysis.

**Fig. 1:**
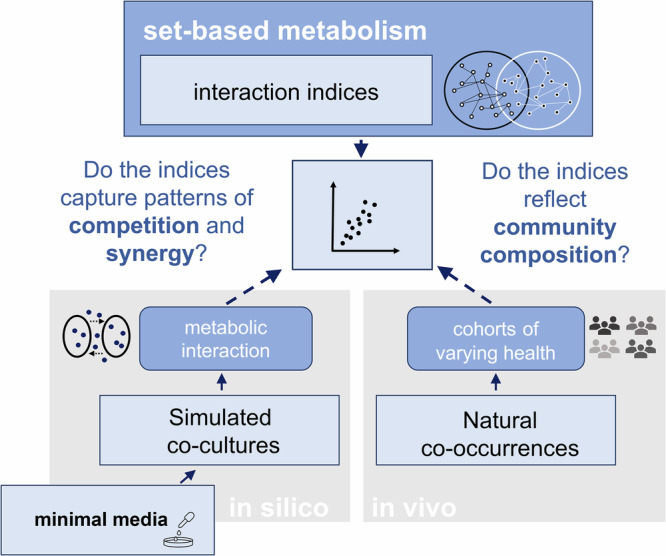
Structure of this study. We compute interaction indices from GEMs of 73 gut microbial species. These indices are evaluated as indicators of competition and synergy via an in-silico approach (left branch). We simulate co-cultures of the 73 species in the agent-based modeling framework BacArena. Flux-balance analysis in cobrapy (Version: 0.26.3) was used to determine the corresponding minimal media. The extracted patterns of pairwise cross-feeding and shared feeding are correlated with the interaction indices. In an in-vivo approach (right branch), we explore the extent to which the indices reflect the community composition across fecal samples of 124 individuals. Correlations with the derived co-occurrence data are computed for groups of varying body mass index and health status. The figure was created using PowerPoint.

Resorting to an in vivo study, we try to answer the question if our indices reflect community composition. To this end, we correlate the indices with natural co-occurrences of the microbial species from fecal samples of 124 individuals. Metadata on the individuals’ body mass index and bowel diseases allows us to examine the performance of the indices in sub-cohorts of varying health status. Figure 1 summarizes the structure of our investigation.

### Symmetric and asymmetric overlap in metabolic networks as an indicator of competitive and synergistic potential

**Fig. 2:**
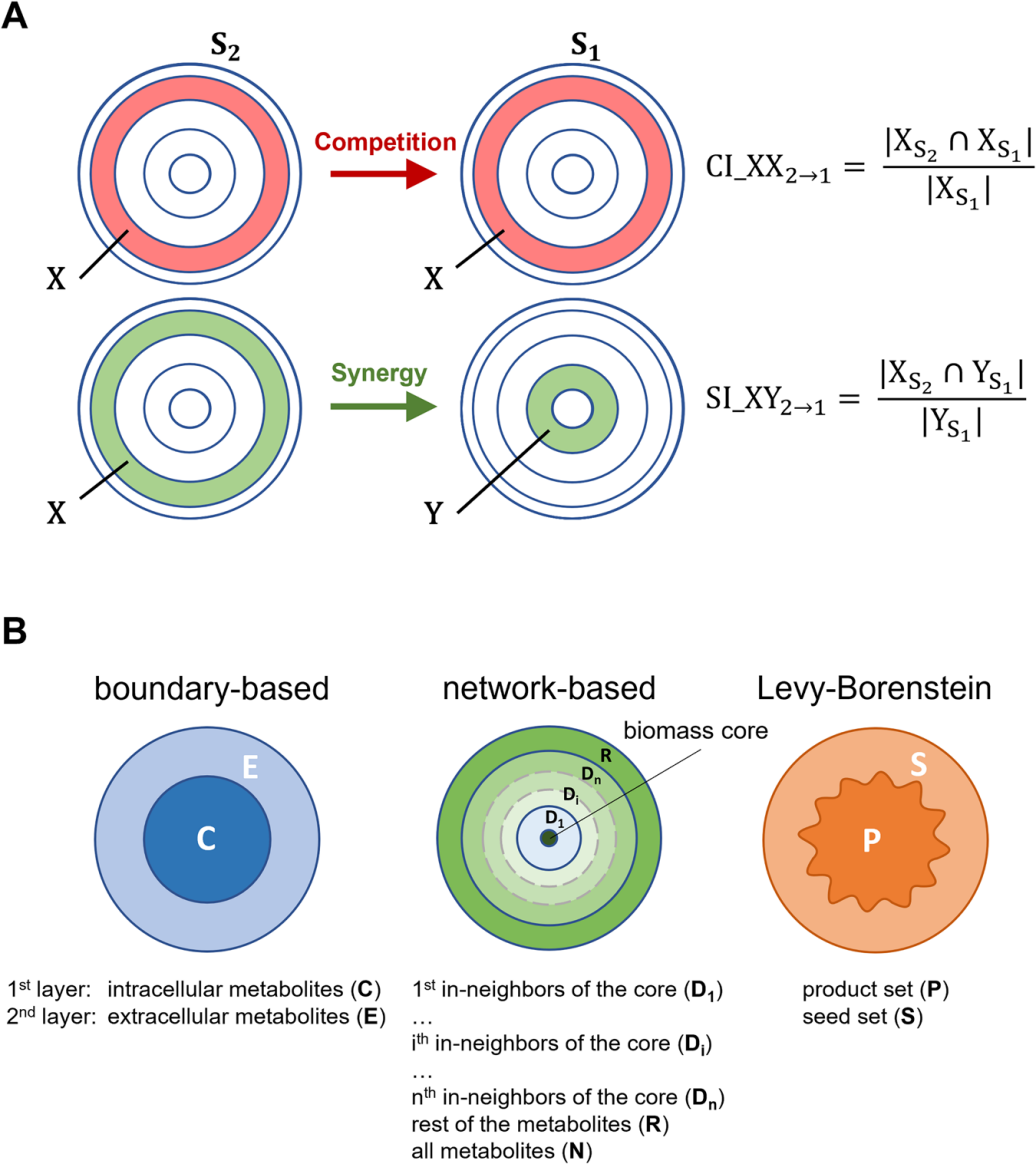
Definition of metabolic interaction indices (schematic). **A** The metabolic networks of two species S2 and S1 are conceptualized as circles, with orbits representing the different layers of the network. The competitive potential that S2 exerts on S1 (the *competitive index* CI) is computed as the number of metabolites appearing both in layer *X* of species S2 and the analog layer *X* of species S1, normalized by the total number of metabolites in layer *X* of species S1. The synergistic potential that S1 experiences by S2 (the *synergistic index* SI) is computed as the number of metabolites appearing both in layer *X* of species S2 and in the different layer *Y* of species S1, normalized by the size of layer *Y*. **B** Schematic representation of the layering underlying the different types of indices. The circles depict metabolic networks, with the orbits illustrating different layers. Boundary-based indices are based on a simple classification of metabolites according to their location, which is either intracellular (C) or extracellular (E). For the network-based indices, metabolites are grouped according to their metabolic distance to the *core* of the network (D1, D2,…, R), given by the products of the biomass reaction. Levy-Borenstein distinguishes two layers, the product set (P) and the seed set (S). The curly inner circle illustrates the elaborate topological distinction between the two sets. The figure was created using PowerPoint.

We derive directed indicators of competitive and synergistic potential between two species based on their metabolic networks. In its simplest form, competition is an overlap in resource usage. Intuitively, this translates into an overlap of the uptake reactions of the two species and, by extension, into an overlap of the metabolites in the same region in their metabolic networks. To operationalize this idea, we divide a metabolic network into non-overlapping node sets, which we denote as *layers* (see Fig. 2A). We measure the competitive potential exerted by one species on another as the intersection of metabolites in equivalent layers of their respective metabolic networks. Different ways of defining layers will result in different versions of this index.

With respect to synergy, it is more challenging to construct a suitable proxy from the metabolic representation. In its most basic form, synergy is an organism’s usage of resources provided by another organism. Structurally, this translates into an overlap of distinct metabolic layers: Most radically, synergy is associated with the secretion layer of one organism overlapping with the uptake layer of another organism. Here, we extend this basic notion to any two distinct metabolic layers and define the synergistic potential that the first species experiences by the second species as the asymmetric overlap of these two layers.

These definitions are summarized in Fig 2A. In the following, we will refer to these quantities as *formal* competitive and synergistic indices and evaluate their suitability as indicators of the respective interaction. For simple graphic representations of the indices defined below, refer to Supplementary Tables 1 and 2.

#### Boundary-based indices

A microbial cell naturally provides two layers, namely the “inside” and the “outside” of the cell, separated by the cell wall as a boundary. In the sense of the metabolic network, those regions translate to the intra (C)- and extra-cellular (E) metabolites. While intra-cellular metabolites can only appear in the cytoplasm of a microbial cell, extra-cellular metabolites can only exist in the environment of the cell, from where they can be taken up or into which they can be released. This way of defining the layers does not take into account the network structure, instead it is solely based on the distinction between inside and outside of a cell (‘boundary-based’) and therefore both simple and intuitive (cf. Fig 2B and Supplementary Fig. 1A).

#### Network-based indices

This more refined version of an index exploits the rich structure of the metabolic network. With regard to the growth of a microorganism, we consider the products of the biomass reaction as the core of its metabolic network (compare^26^). Intuitively, the metabolites, which are “closer” to the core in the metabolic network, contribute more directly to the biomass production and thereby to the growth of an organism. With increasing distance to the core, the contribution of a metabolite to biomass production becomes more and more indirect. For the network-based indices, we define layers on the basis of metabolite distance to the core. More precisely, we define the *metabolic distance* of a metabolite as the length of the shortest directed path from the metabolite to any of the core elements in the metabolic network (cf. Fig 2B). Consequently, the first layer is given by all the metabolites that have a directed edge to at least one of the core metabolites in the network. This layer is denoted by D_1_ and represents all metabolites that can be directly transformed to a core metabolite through a reaction. The second layer (D_2_) contains all metabolites for which there exists a directed path of length two to at least one of the core metabolites. Those metabolites which do not have a directed path to any of the core metabolites, the “rest”, form the layer R (see Supplementary Fig. 1B for an exemplary presentation of this layering). All metabolites occurring in the network are aggregated in the layer N. For the sake of completeness, we additionally construct an index Edge. The name already indicates that this index deviates from our general definition in taking not only nodes, but also edges in the metabolic network into account (see *Methods*). Refer to Supplementary Data 01 for all interaction indices calculated among the species pairs.

We compare our network-based indices to a purely topological concept of layers, the so-called k-shells^27,28^. A k-shell decomposition organises nodes into layers of increasing connectivity via iterative removal of low-degree nodes. The decomposition is conducted by assigning all nodes of degree < 1 to a shell, pruning them from the network, updating node degrees, and repeating this step for nodes of degree < 2, and so on. In our notation, k_1_ denotes the innermost shell, corresponding to the most densely connected subgraph. See *Methods* for details on the layering procedure. Note that the expected comparability between those two types of indices builds on the fact that the routing of all distances through the hubs of a network (and hence a lack of sensitivity of distance patterns to the selected core) is a typical feature of networks with a broad degree distribution (see e.g., ref. ^29^).

#### Levy-Borenstein indices

In ref. ^17^, Levy and Borenstein present network-based interaction indices, which can be understood as a special case of our general framework. They divide a metabolic network into a *seed set* (S) and a complementary *product set* (P). Topologically, they define a node as a seed node if it belongs to a strongly connected component that has no incoming edges and at least one outgoing edge. Hence, the seed set of a species contains all compounds that cannot be produced by the species, but only acquired exogenously (see ref. ^30^). All remaining nodes are classified as product nodes. The seed and product set can be perceived as two layers of the metabolic network. See ref. ^17^ for a simple schematic representation and Supplementary Fig. 1C for an exemplary presentation of the S and P layer in a metabolic network. In accordance with our general framework, Levy and Borenstein define the competitive index between two species as the intersection of their seed sets, and the complementarity (synergistic) index as the intersection of the product set of the second species and the seed set of the first species (cf. Fig. 2). Our results are based on the original values of the indices reported by Levy and Borenstein in ref. ^17^.

Note that due to their sizes, both the product set P and the set of intracelluar metabolites C on average make up more than 67% of the complete set of metabolites N (compare Supplementary Fig. 2). Interestingly, the product set P has an average overlap of more than 67% with the set of intracellular metabolites C, while the product set’s complement, the seed set S, has the highest intersection with the set of extracellular metabolites E (> 20%). This follows Levy-Borenstein’s intuition of the seed set as comprising the metabolites that have to be externally received by a species.

### Competition in simulated microbial communities is best explained by overlap in the middle network layers

In order to validate our indices, we compare them to the metabolic activity in simulated co-cultures of 73 microbial species of the human gut microbiome. To this end, we run pairwise simulations using the agent-based community modeling tool BacArena (*Methods* and Supplementary Note 2). This in-silico approach allows us to extract the exact composition and amount of nutrients being consumed, secreted, and exchanged among the individuals of different species. We use the proportion of the overlap between the nutrients being consumed by two different species (*shared feeding*) as a measure of the competition that one of them experiences by the other one. Analogously, we consider the overlap between the nutrients being consumed by one species and those being secreted by another species (*cross feeding*) as a measure for the synergy that the first species experiences by the second one. We test the applicability of our different indices to serve as indicators of competition and synergy by correlating them with pairwise shared feeding and crossfeeding for ten communities of 25 randomly chosen species each (see Supplementary Note 2).

**Fig. 3:**
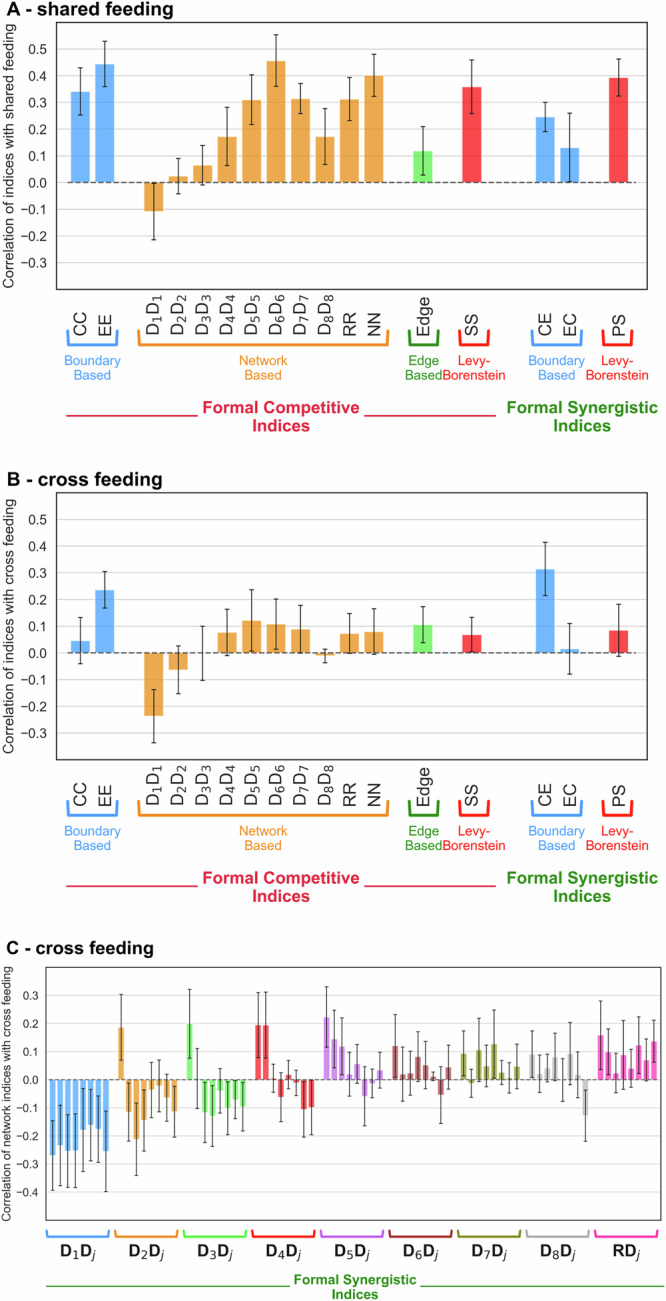
Performance of interaction indices as indicators of simulated metabolic activity among 73 microbial species of the human gut microbiome. Mean and standard deviation are computed across ten random sets of 25 species each (see Supplementary Note 2). **A** Correlations between formal competitive and synergistic indices and shared feeding. **B** Correlations between formal competitive and synergistic indices and crossfeeding. **C** Correlations between network-based formal synergistic indices and crossfeeding. The exact correlation results can be found in Supplementary Data 02.

Except for D_1_D_1_, all mean correlations of formal competitive indices with shared feeding are, as expected, positive (Fig. 3). Furthermore, all formal competitive indices yield higher correlations with shared feeding than with crossfeeding (compare Fig 3A, B). The highest correlation (*c* = 0.46) is reached by the network-based index D_6_D_6_, measuring the overlap between the middle network layer D_6_ of two species (Fig. 3A). Interestingly, starting from a negative correlation for the central network layer D_1_, correlations increase up to D_6_, and start decreasing again from there on to the further peripheral layer of D_8_. A slightly lower correlation than for D_6_D_6_ is given by the boundary-based index EE (*c* = 0.44), comparing the set of all extra-cellular metabolites of two metabolic networks. The simple overlap of all metabolites occurring in the species’ metabolic networks (NN) still yields a correlation of *c* = 0.4. Interestingly, extending the comparison to the edges of the metabolic networks (index Edge) decreases the correlation to *c* = 0.12. Levy-Borenstein’s comparison of the species’ seed sets (SS) yields a moderate correlation of *c* = 0.36.

### A simple comparison of intra- and extra-cellular metabolites is the best indicator for in-silico synergy

Compared to the correlations of formal competitive indices with shared feeding, the mean correlations of formal synergistic indices with crossfeeding are markedly lower (Fig. 3B, C). Furthermore, in contradiction to their formal definition, both Levy-Borenstein’s synergistic index PS and the boundary-based synergistic index EC correlate higher with shared feeding (*c* = 0.39 and *c* = 0.13, respectively) than with crossfeeding (*c* = 0.08 and *c* = 0.01, respectively). Interestingly, the reversed boundary-based index CE, measuring the overlap between the intra-cellular metabolites of species S2 with the extra-cellular metabolites of species S1 (compare Fig. 2A), shows the expected behavior: it correlates more strongly with crossfeeding (*c* = 0.31) than with shared feeding (0.25).

Among the network-based formal synergistic indices, the indices D_*i*_D_1_, comparing layer D_*i*_ with layer D_1_ of species S2 and S1, respectively, clearly stand out, with D_5_D_1_ reaching a mean correlation of *c* > 0.2 (Fig 3C). Matching their formal definition, they only correlate marginally with shared feeding (see Supplementary Fig. 4). This pattern more or less dissipates between layers D_6_ and D_8_. Among RD_*j*_, RD_1_ yields the highest correlation again. In contrast to the correlations between competitive indices and shared feeding, correlations between synergistic indices and crossfeeding show large standard deviations across the ten random sets of species.

### Competitive and synergistic indices reveal strong contrasting patterns in gut microbiome

After assessing the relationship between interaction indices and metabolic activity in in-silico co-cultures of human gut microorganisms, we explore their relation to in-vivo co-occurrence patterns of these very species^17^ (see *Methods*). Note that both shared- and cross-feeding show only weak correlations with co-occurrence patterns (compare Supplementary Fig. 5). The data encompasses multiple cohorts of individuals differing in BMI (body mass index) and health status (with or without IBD (inflammatory bowel disease))^31^. We consider correlations between indices and co-occurrence data based on samples of all cohorts, as well as for four subgroups of different health status: Healthy-Lean, Healthy-Obese, IBD-Lean, and IBD-Obese.

#### Overlap in equivalent network regions is a robust indicator of co-occurrence

**Fig. 4:**
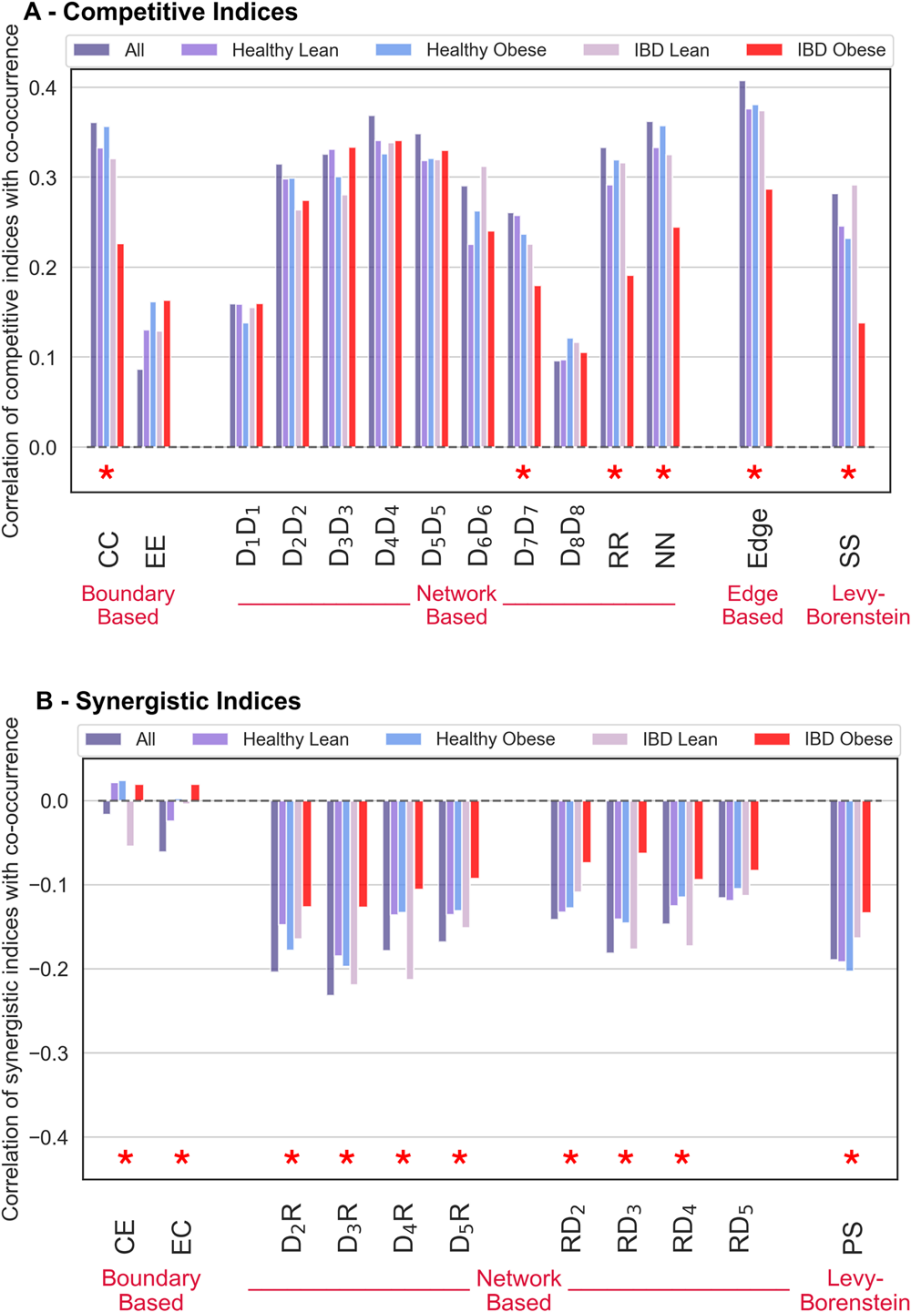
Performance of interaction indices across subgroups of patients with diverse health statuses. **A** Correlation between co-occurrences and formal competitive indices. **B** Correlation between co-occurrences and formal synergistic indices that show the highest correlation in Supplementary Figures 6-10. Asterisk (*) associated with an index represents a significant difference (*p* < 0.05) in correlations of subgroup IBD-obese compared to the subgroup containing all individuals across the ten species sets (see Supplementary Data 03 and 04 for comparison with other subgroups). The exact correlation results can be found in Supplementary Data 02.

The correlations of all the competitive indices with the microbial co-occurrences are positive (Fig. 4A). Considering results for all cohorts combined, the index with the highest mean correlation is the Edge index (*c* = 0.41), which measures the overlap of edges between the two metabolic networks, followed by the network-based index D_4_D_4_ (*c* = 0.37), which assesses the overlap between the middle layers D_4_ of both networks. The boundary-based index CC and the network-based index NN, which measures the overlap of all nodes of the two metabolic networks, trail slightly behind, with a mean correlation of *c* = 0.361. Levy-Borenstein’s seed set index SS yields a correlation of *c* = 0.281 only. Notably, similar to the correlations with simulated metabolic activity, the correlations of co-occurrences and network-based indices follow a trend in which they initially increase up to a certain middle layer (here D_4_) and then decrease up to the most peripheral (but still directly connected) layer D_8_. The (in a directed sense unconnected) layer R exhibits a comparatively high correlation again (*c* = 0.33). Note that for k-shell layering, we observe a similar performance as for our biologically-informed network-based layers (see Supplementary Fig. 11). The highest correlations are consistently achieved by a middle layer, the 2-shell, and they are close to the correlations of index D_4_D_4_, the best-performing network-based index (see Supplementary Data 02).

Overall, the competitive indices are remarkably stable in their positive correlation with co-occurrence data, regardless of the selected layering method.

#### Overlap of middle and peripheral network layers shows the strongest negative correlation with co-occurrence data

Notably, nearly all correlations between co-occurrences and the formal synergistic indices are negative, strongly contrasting the competitive indices (Fig. 4A, B, Supplementary Figs. 6-10). (Note that, given the large number of synergistic network-based indices, we present only the top-performing ones in Fig. 4B). In absolute values, however, the correlations with synergistic indices are lower than those with competitive indices. The intersection of the middle network layers (D_2_, D_3_, D_4_, D_5_) with layer R yields the highest negative correlation. While Levy-Borenstein’s index PS exhibits a high negative correlation with the co-occurrences, the basic boundary-based indices (CE, EC) exhibit very poor correlations. Interestingly, the synergistic k-shell indices k_1_k_4_ and k_4_k_1_ show the highest significant negative correlation (see Supplementary Data 02 and 04), where k_1_ is the innermost and k_4_ (analogous to layer R) is the outermost layer.

As we have already seen for correlations with in-silico metabolic activity, the standard deviations are significantly higher in the case of synergistic indices than in the case of competitive indices (compare Supplementary Figs. 6–10).

### Predictive power of interaction indices varies with health status of patients

The comparison of correlations for each subgroup (Fig. 4) reveals an interesting pattern: While correlations for the groups of healthy-lean, healthy-obese, and IBD-lean individuals are close to each other across most indices, the subgroup of IBD-obese individuals is a clear outlier.

Across the ten randomly sampled species sets, the absolute correlations between co-occurrence patterns in the IBD-obese subgroup and the competitive indices were significantly lower than those observed in all other health subgroups. The significant differences among subgroups were supported by one-tailed paired tests on Fisher-transformed correlation values (see Methods). Comparison of the subgroup ‘IBD-obese’ with ‘all’ showed significant differences for competitive indices SS (*p* = 0.0004), CC (*p* = 0.0002), RR (*p* = 0.0014), NN (*p* = 0.0005) and Edge (*p* = 0.0009) (see Supplementary Data 03), and all synergistic indices shown in Fig. 4B, except RD_5_ (see Supplementary Data 04).

## Discussion

In this study, we present a generalized metabolic model of microbial interactions. The suggested framework is based on the simple principle of overlapping the same/different subsets of two metabolic networks to gain indices of competition/synergy of two organisms. Starting from the radically simple sets of intra- and extracellular metabolites, we move towards a more complex, network-based set structure, building on the distance of metabolites from the “biological core” of the network. For validation, we correlate the indices of 73 prevalent gut microbial species with in-silico metabolic activity and in-vivo co-occurrences. Remarkably, the simplest of all indices are always among the best indicators, explaining competition, synergy, and community composition to a greater extent than more sophisticated partitionings like Levy-Borenstein’s seed and product set^17^. After all, microbial cohabitation seems to be – in astonishingly large parts – a simple game of needs. But our results also illustrate that the rules of this game can change. There is a strong discrepancy in the performance of the indicators between IBD-obese and healthy cohorts. This observation aligns with the growing evidence that gut bacterial interaction networks can be strongly context-dependent^32^.

Based on this first evidence, correlation of microbial co-occurrence patterns with our indices could be envisioned as a ‘biomarker’ for assessing the health status of a microbiome.

The performance of single indicators varies strongly between in-silico metabolic measures and in-vivo co-occurrence patterns. These discrepancies emphasize that community composition is driven by more than pure metabolism. For example, simple overlap between two sets of extracellular metabolites (index EE) is one of the strongest indicators of shared feeding, while overlap between the intracellular metabolites of one species and the extracellular metabolites of another species (index CE) yields the highest correlation with cross-feeding. These results support the intuition that the set of extracellular metabolites is highly informative about species’ feeding patterns. However, both index EE and index CE yield particularly low absolute correlations with co-occurrences, showing their small value as indicators of community composition.

Despite all their differences, both the in-silico metabolism and the in-vivo co-occurrence analysis reveal the same distinctive pattern when it comes to the network-based competitive indices: with growing distance to the network core, correlations increase up to the middle layers (D_6_ for shared feeding, D_4_ for co-occurrences), where they start to decrease again. Hence, the most information on shared feeding patterns and community composition seems to be hidden in those metabolites that do not directly contribute to the biomass reaction but whose metabolic distance is not too large at the same time. Interestingly, the metabolite composition differs the most among the species in these middle layers (see Supplementary Fig. 12), suggesting that the parts of metabolic networks that are shared among most species can be considered as “noise” regarding the question of interaction and co-occurrence.

The performance and diversity of these intermediate layers suggest a potential bow-tie architecture in metabolic networks. Bow-tie architecture is characterized by a narrow middle layer ("knot”) with larger input ("bow”) and output ("tie”) layers, fostering a balance between robustness and efficiency^33,34^. While previous studies have explored bow-tie topologies in genome-scale metabolic models (GEMs) using flux balance analysis^35,36^, their role with respect to microbial interactions and community formation has been insufficiently studied. Furthermore, our framework provides a new approach to systematically define sets of key metabolites, which could serve to reduce the degrees of freedom in GEM analysis or to identify specific targets for biotechnological applications.

In agreement with ref. ^30^, all formal competitive indices in our study show positive correlations with compositional data of the human gut microbiome, while all formal synergistic indices show either marginally low or negative correlations. This pattern indicates that species with similar metabolic profiles tend to co-occur rather than out-compete each other, a phenomenon fostered by the abundance of nutrients in the gut environment. Accordingly, one could argue that these results support the hypothesis that the human gut microbiome is mainly habitat-filtered in contrast to a primarily interaction-shaped microbial community^37^. However, some limitations remain: Firstly, shared feeding and cross-feeding are only aspects of competition and synergy. There is much more to what is commonly understood by those notions, such as pH modulation^38^, toxin production, and secretion^39,40^. Furthermore, our approach does not account for non-pairwise effects or the influence of spatial organization^23^. These limitations supposedly add to the fact that correlations overall do not exceed values of 0.5 and should be overcome in future studies. Promising starting points for such studies are provided by the more complex but constrained and well-studied microbial community models SIHUMI^41^ or OligoMM^42^.

Secondly, it remains to be shown that synergistic indices indeed correlate positively with co-occurrences in interaction-driven communities. Otherwise, a lack of correlation can hardly be interpreted as the absence of strong synergistic mechanisms. This effort is further complicated by the fact that several synergistic indices, among them the established Levy-Borenstein index PS, correlate more strongly with shared feeding than with cross-feeding, highlighting the conceptual challenge to adequately capture synergistic interaction. It is intuitively understandable that the composition of the human gut microbiome, being rich in nutrients, is strongly driven by habitat filtering^9,43,44^. Hence, more scarce habitats, such as the lungs or the upper respiratory tract, could serve as suitable case studies for interaction-driven microbial communities^45,46^.

Moreover, while the majority of indices show a big difference when applied to the IBD obese cohort, the difference for most *D*_*i*_*D*_*i*_ is negligible. We hypothesize that these differences between categories of indices could hint at *functional* differences between metabolites. In the case of IBD obese, the observation that *D*_*i*_*D*_*i*_ indices are uninformative could indicate that the functional differences are about the processing of the environment, rather than the internal metabolic processes.

Going beyond microorganisms and their metabolic networks, the principle of comparing the same and different functional levels to detect competition and synergy could be translated to and explored in other complex systems. Whenever the functional potential is hierarchically structured, such as in trophic networks, levels could be identified based on their distance from the functional core, analogously to the network-based indices.

## Methods

### Species and co-occurrence data

Levy and Borenstein compare their indices to natural co-occurrences of 154 microbial species that were found to be prevalent (present in at least one individual at ≥ 1 % coverage) across fecal samples of 124 individuals from Denmark and Spain, varying in Body Mass Index (either lean or obese) and health status (either healthy or diagnosed with a chronic inflammatory bowel disease (IBD))^17,31^. They determine the co-occurrence between every pair of species via the abundance-based continuous Jaccard similarity index. We compare our indices to the co-occurrences and indices given in ref. ^17^. However, we restrict our analysis to the subset of 73 species whose metabolic models are available on the Virtual Metabolic Human database vmh.life (Supplementary Data 05). This restriction is due to the fact that BacArena, the community modeling software used in this study, is based on vmh models^21,25^.

We create ten random subsets of 25 species each (Supplementary Data 05) by repeatedly applying the R base function sample without replacement to the set of 73 species. Each of the 73 species is included in at least one of the ten subsets, and each pair of subsets overlaps in twelve species maximally. We use those subsets for stability testing.

### Reconstruction of the metabolic networks

To reconstruct the metabolic networks, we obtained genome-scale metabolic reconstructions (GEMs) that were semi-automatically generated for human gut microorganisms from AGORA (assembly of gut organisms through reconstruction and analysis)^47^, version 1.03 (June 22, 2024), via the Virtual Metabolic Human (VMH) database (vmh.life)^25^. For each species, the list of reactions was extracted from the respective model. Using this reaction list, we constructed a directed, unweighted network by setting an edge (m1, m2) if a reaction existed in which metabolite m1 was a reactant and metabolite m2 was a product. In line with the methodology described in ref. ^30^, the glycans and the top 3% of currency metabolites were removed from the resulting networks. Glycans are complex compounds that are inconsistently annotated in literature and are not central to the metabolic processes^48–50^; removing glycans results in more robust and comparable networks across species. Currency metabolites are ubiquitous and highly connected compounds, e.g., ATP/ADP, NAD^+^/NADH, H_2_O etc. These compounds participate in many reactions and can distort network topology by creating shortcuts between unrelated pathways^51,52^. A detailed description of the procedure is provided in Supplementary Note 1.

### Notes on the computation of the interaction indices

In the following, we denote by *G*_*S*_ = (*V*_*S*_, *E*_*S*_) the metabolic network of species *S*, consisting of the set of nodes (metabolites) *V*_*S*_ and the set of edges (reactions) *E*_*S*_. The computation of the network-based indices between species *S*_1_ and *S*_2_ requires the division of their metabolic networks ${G}_{{S}_{1}}=({V}_{{S}_{1}},{E}_{{S}_{1}})$ and ${G}_{{S}_{2}}=({V}_{{S}_{2}},{E}_{{S}_{2}})$, respectively, into layers based on the metabolic distance ${d}_{v,{S}_{i}}$ associated with each metabolite $v\in {V}_{{S}_{i}}$ (cf. *Results*). All metabolites without path to the network’s core are subsumed in the additional layer R. Hence, by definition, the number of layers for a network ${G}_{{S}_{i}}$ is given by

$$\max \left({d}_{v,{S}_{i}}\,| \,v\in {V}_{{S}_{i}}\right)+1.$$

However, the maximal metabolic distance can differ among species. Therefore, whenever we are calculating network-based indices within a community of species {*S*_*i*_ ∣ *i* ∈ *I*}, we use the smallest number of layers as the community-wide upper threshold, adding all metabolites with metabolic distance larger than the minimal maximal metabolic distance

$$\min \left(\max \left({d}_{v,{S}_{i}}\,| \,v\in {V}_{{S}_{i}}\right)\,| \,i\in I\right)$$

to a species’ R-layer. This procedure results in the same number of layers for all species.

The special index ‘Edge’ is computed by determining the normalized overlap of directed edges between the metabolic networks of two species. More precisely, for two species *S*_1_ and *S*_2_, we consider the sets of directed edges ${E}_{{S}_{1}}$ and ${{\rm{E}}}_{{S}_{2}}$, with each edge being identified by its ordered start and end point metabolites (*m*_1_, *m*_2_), and compute

1 $${E}_{2\to 1}=\frac{| {E}_{{S}_{2}}\cap {E}_{{S}_{1}}| }{| {E}_{{S}_{1}}| }$$

following the concept illustrated in Fig. 2A.

The computation of k-shell indices makes use of the core_number function in NetworkX^53^. This method is based on the k-core decomposition of a network, in which nodes are iteratively removed when their degree drops below the current value of k. At each step, the node with the lowest degree is removed, and the degrees of its neighbors are updated accordingly. The core number of a node is defined as the highest value k for which the node remains in the corresponding k-core, i.e., a maximal subgraph in which all nodes have degree at least k. Here, to enable comparison with network-based indices, we re-index k-shells in reverse order, k_1_, k_2_,…, such that k_1_ denotes the innermost shell corresponding to the highest k-core.

### Simulating co-cultures of microbial species in BacArena

We use BacArena^21^, an R-based community modeling tool combining FBA and individual-based modeling, to simulate ten co-cultures of 25 microbial species of the human gut each (Supplementary Data 05) (R version 4.2.1). In the spatially and temporally resolved simulations, the metabolism and growth rate of an individual organism are determined by parsimonious FBA on the corresponding GEM provided by the Virtual Metabolic Human database^25^. Constraints are given by the concentration of nutrients in the respective grid cell at the respective time point. Nutrient concentration in each cell is updated according to secretion, consumption, and diffusion, and the duplication rate follows from the growth rate.

We define *shared feeding* of species S1 with respect to species S2 as the total amount of nutrients being consumed by both S1 and S2, normalized by the total amount of nutrients being consumed by S1. We define *crossfeeding* from S2 to S1 as the total amount of nutrients being both produced by S2 and consumed by S1, normalized by the total amount of nutrients being consumed by S1. We run each simulation for a variety of *richness levels*, denoting the proportion of the possible medium that is added to the minimal medium to create the initial medium. Moreover, we conduct ten repetitions per richness level, with the set of nutrients being randomly drawn anew in each repetition, and average the derived interaction measures across these repetitions.

See Supplementary Note 2 for details on the simulations.

### Correlating interaction indices with simulated metabolic activity and co-occurrences

For each random set and each richness level, we compute Spearman and Pearson correlation between interaction indices and shared- and crossfeeding values of all directed pairs of species. If the *p*-values of both Spearman and Pearson correlation fulfill *p* < = 0.05 and the correlation coefficients are either both positive or both negative, we assign the (absolutely) larger of the two coefficients as the correlation to the respective random set and richness level. If those conditions are not fulfilled, we set the correlation to zero. To assess overall correlations, we first average correlations across richness levels and, in a second step, compute the mean and standard deviation of the correlations across the ten random sets.

The procedure for correlating interaction indices with co-occurrence data is largely analog, however, it requires one additional pre-step. While interaction indices are by definition asymmetric, yielding two values per pair of species, co-occurrence values are symmetric. Following^17^, we average the two interaction indices of a species pair (${\overline{XY}}_{{S}_{1}{S}_{2}}=\mathrm{mean}(X{Y}_{{S}_{2}\to {S}_{1}},\,X{Y}_{{S}_{1}\to {S}_{2}})$) before correlating this mean with the respective co-occurrence value. Following this averaging, the indices

$${\overline{XY}}_{{S}_{1}{S}_{2}}=\frac{\frac{| {X}_{{S}_{2}}\cap {Y}_{{S}_{1}}| }{| {Y}_{{S}_{1}}| }+\frac{| {X}_{{S}_{1}}\cap {Y}_{{S}_{2}}| }{| {Y}_{{S}_{2}}| }}{2}$$

and

$${\overline{YX}}_{{S}_{1}{S}_{2}}=\frac{\frac{| {Y}_{{S}_{2}}\cap {X}_{{S}_{1}}| }{| {X}_{{S}_{1}}| }+\frac{| {Y}_{{S}_{1}}\cap {X}_{{S}_{2}}| }{| {X}_{{S}_{2}}| }}{2}$$

differ only as a consequence of disparities in the sizes of the respective layers; in the case of equal layer sizes, the two indices are identical.

### Statistical comparison of correlations for different health subgroups

To compare whether the correlation between the metabolic indices and co-occurrences differs significantly between cohorts of different health states, we perform a one-sample statistical test on paired Fisher-transformed correlation values across the ten sets of species.

For the comparison between two health states, e.g., IBD-obese and IBD-lean, the correlation values were first transformed using Fisher’s r-to-z transformation: *z* = *tanh*^−1^(*r*). In a second step, we computed the pairwise differences *d* = *z*_*IBD*−*obese*_ − *z*_*IBD*−*lean*_ for each index. We checked the normality of the distribution of pairwise differences using the Shapiro-Wilk test. If the normality assumption was not rejected (*p* > 0.05), a one-sample t-test was applied to *d*, otherwise, we used the Wilcoxon signed-rank test. For the one-tailed comparison, the alternatives were chosen based on the direction of the hypothesis (for competitive indices, IBD-obese was expected to be lower than others, and for synergistic indices, higher). All tests were performed using scipy.stats^54^ (Version: 1.16.0) in python. All test results for the competitive and synergistic indices can be found in Supplementary Data 03 and 04, respectively^25,30,47,55–58^.

## Supplementary information

Supplementary Information

Supplementary Data 01

Supplementary Data 02

Supplementary Data 03

Supplementary Data 04

Supplementary Data 05

Supplementary Data 06

## Data Availability

The datasets generated and/or analyzed during the current study are available in the Supplementary and in the GitHub repository: Metabolic_set_theory under https://github.com/Jojo6297/Metabolic_set_theory.git.

## Code availability

The code used to generate the results of the current study are available in the GitHub repository: Metabolic_set_theory under https://github.com/Jojo6297/Metabolic_set_theory.git.
