# Supplementary material for: Metabolic set theory: a generalized model of microbial interactions
Source: NPJ Syst Biol Appl. 2026 Jul 1;12:94. doi: 10.1038/s41540-026-00774-4 (PMC13324171; doi:10.1038/s41540-026-00774-4)
Supplement: Supplementary file 1 — Supplementary Information [file 41540_2026_774_MOESM1_ESM.pdf]

Metabolic set theory: a generalized model of microbial interactions

Jyoti Jyoti, Hannah Zoller*, Wolfgang zu Castell, Marc-Thorsten Hütt

* E-mail: hannah.zoller@su.se

Supplementary Notes

Supplementary Note 1: Reconstruction of the metabolic networks

The reconstruction of networks conducted in this study closely parallels the methodology presented in [1]. However, while the authors of [1] used the KEGG database [3] to obtain a list of metabolic reactions for each species, we use a collection of genome-scale metabolic reconstructions semi-automatically generated for human gut microorganisms, i.e., AGORA (assembly of gut organisms through reconstruction and analysis) [5], version 1.03 (June 22, 2024) from the Virtual Metabolic Human (VMH) database website vmh.life [7].
We work with the VMH website because the BacArena framework utilizes vmh models. Furthermore, the genome-scale metabolic models (GEMs) on vmh.life specify the location of the metabolites in the system, distinguishing between the cytosol [c] and the extracellular space [e]. Only metabolites in the [e] compartment can be exchanged among the species and hence, we consider the information regarding the compartmentalization of metabolites crucial for addressing the question of microbial interactions. Accordingly, compounds with different compartment suffices will occur as different nodes in our network. In particular, this distinction forms the foundation of the boundary-based indices. However, this provides a challenge when comparing metabolites between the C and E sets, as the same compound may appear with different suffixes. In this specific case, comparisons are performed using only the core metabolite identifiers, disregarding the compartment specific suffixes.
For each species, the list of reactions is extracted from the model. In a first step we exclude all sink and source reactions (no products or no reactants, respectively). In a second step, we create a directed, unweighted network by considering all metabolites occurring in the list as nodes and by adding edges according to the following rule: If there exists a reaction in which metabolite m_1 is one of the reactants and metabolite m_2 one of the products, we add a directed edge from m_1 to m_2. Reversible reactions yield edges in both directions each.
Following the procedure in [1], we apply two filtering steps to the resulting networks. At first, all glycans are removed from the network due to their structural complexity and their limited role in the core metabolic processes. In a second step, we reduce the number of currency metabolites in the network. To this end, the authors of [1] restrict their network from the beginning to the "main reactions" as provided by the KEGG database. To follow a more transparent selection criterion, we follow the common practice of removing supposed currency metabolites from the created network by eliminating the top 3% nodes with the highest degree [4, 6]. In the case of the network-based indices, where biomass reaction products form the network core, the removal of currency metabolites frequently results in the elimination of all core metabolites. In this case, to ensure accurate calculation of the indices, the metabolite 'biomass[c]' is retained.
For additional evaluation and visualization at the level of metabolic layers, rather than solely at the level of indices, we also compute Levy-Borenstein-like seed and product sets on metabolic networks generated using GEMs from the VMH database. These reconstructed layers, referred to as "Levy-Borenstein imitation" layers, are included only in the supplementary data (Supplementary Figures 1C, 2 and 12). To adhere as closely as possible to the procedure described in [1], we introduce an additional filtering step during network reconstruction: removal of small (< 10 nodes) weakly connected components (WCC) that are detached from the largest weakly connected component. Based on the resulting network, we compute seed and product set. This filtering step is not applied to the boundary-based and network-based layers in general, it is used only in Figure S1 to facilitate a visually consistent comparison of network structures.

Supplementary Note 2: Simulating co-cultures of microbial species in BacArena

We simulate the development of two-species communities of initially five individuals each on a two-dimensional grid of size 10x10. The initial nutrient composition in one simulation consists of a certain percentage (*richness level*) of all nutrients which can be consumed by at least one of the two species (the *possible medium*), and the union of the *minimal media* of the two microbial species (Supplementary Data 02). The minimal medium of a species represents a minimal set of nutrients that is required for a certain growth rate. Here, we only want to ensure a non-zero growth rate. We determine these minimal media for each of the 73 species in a two-step approach. In the first step, we identify a set of nutrients ensuring a growth rate of 0.001 via the minimal_medium() function from COBRApy [2] (Version: 0.26.3). In the second step, we iteratively remove nutrients from the medium, check via FBA if the optimal growth rate falls below 0.0001 and, if not, exclude them from our minimal medium.

A simulation is stopped after ten time steps. Making use of BacArena's `findFeeding3()`, we extract and sum up the amount at which each metabolite is being consumed or produced by each species as long as less or equal fifty percent of the grid cells are occupied by microorganisms. The condition reduces spatial effects, which naturally intensify with the filling level of the grid. We translate the extracted metabolic information into two measures of interaction. We define *shared feeding* of species S1 with respect to species S2 as the total amount of nutrients being consumed by both S1 and S2, normalized by the total amount of nutrients being consumed by S1. We define *crossfeeding* from S2 to S1 as the total amount of nutrients being both produced by S2 and consumed by S1, normalized by the total amount of nutrients being consumed by S1. Note that we consider the natural logarithm of the consumption/production of each metabolite to moderate the dominance of only a few, large fluxes.

For each pair of species, we run simulations for richness levels of $0.1, 0.2, 0.3, \ldots, 0.9$, i.e. across the simulations, the initial medium ranges from the minimal media plus randomly drawn 10% of the possible medium to 90%. The initial concentration of each metabolite is set to 0.1 fmol/cell. We conduct ten repetitions per richness level, with the set of nutrients being randomly drawn anew in each repetition, and average the derived interaction measures across these repetitions. This high variability of the initial medium reduces the bias that both nutrient composition and richness of the medium naturally provide when it comes to metabolic activity.

Correlations between shared- and crossfeeding of pairs of species vary between -0.15 and 0.22 across the different sets and richness levels of the medium (see Supplementary Fig. 3).

In order to minimize bias resulting from a specific community composition, we create ten (potentially overlapping) subsets of 25 randomly drawn species each. Results of the pairwise simulations are averaged per subset.

Supplementary Tables and Figures

Supplementary Table 1. Graphical representation of the formal competitive indices: The colored layers in each row denote the intersected sets.

Competitive Indices		
Boundary-based	CC	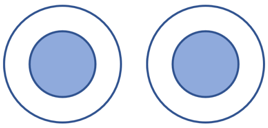
	EE	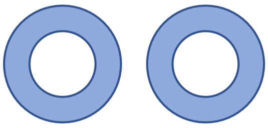
Network-based	D_1D_1	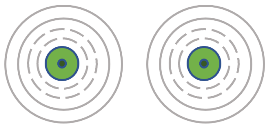
	D_2D_2	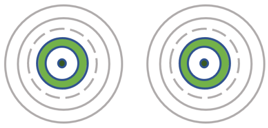
	...	...
	RR	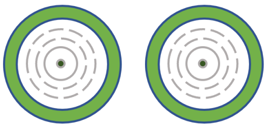
	NN	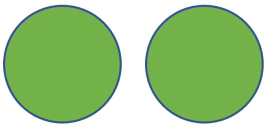
Levy-Borenstein	SS	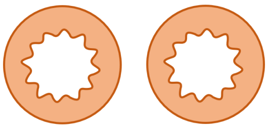
Edge-based	Edge	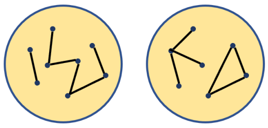

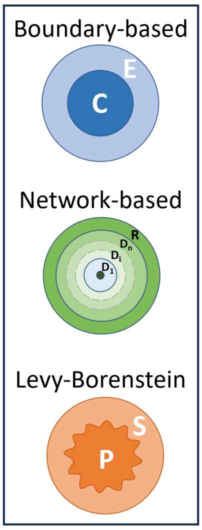

Supplementary Table 2. Graphical representation of the formal synergistic indices: The colored layers in each row denote the intersected sets.

Synergistic Indices		
Boundary-based	CE	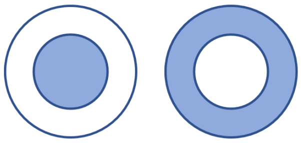
	EC	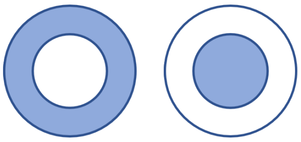
Network-based	D_1D_2	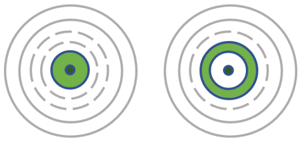
	D_1D_3	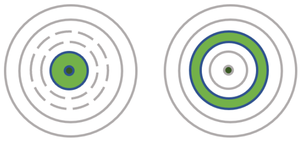
	...	...
	D_1R	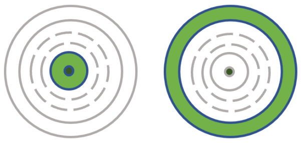
	D_2D_1	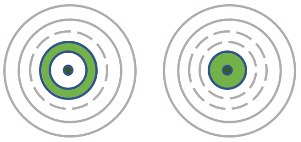
	D_2D_3	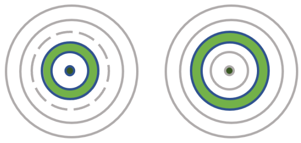
	...	...
	RD_n	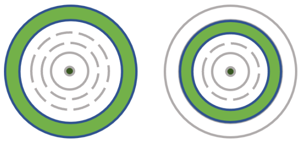
Levy-Borenstein	PS	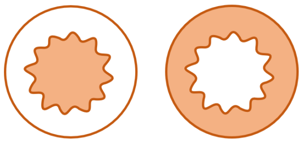

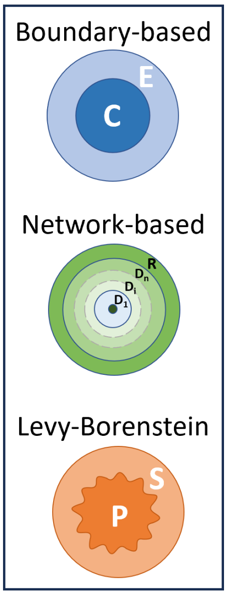

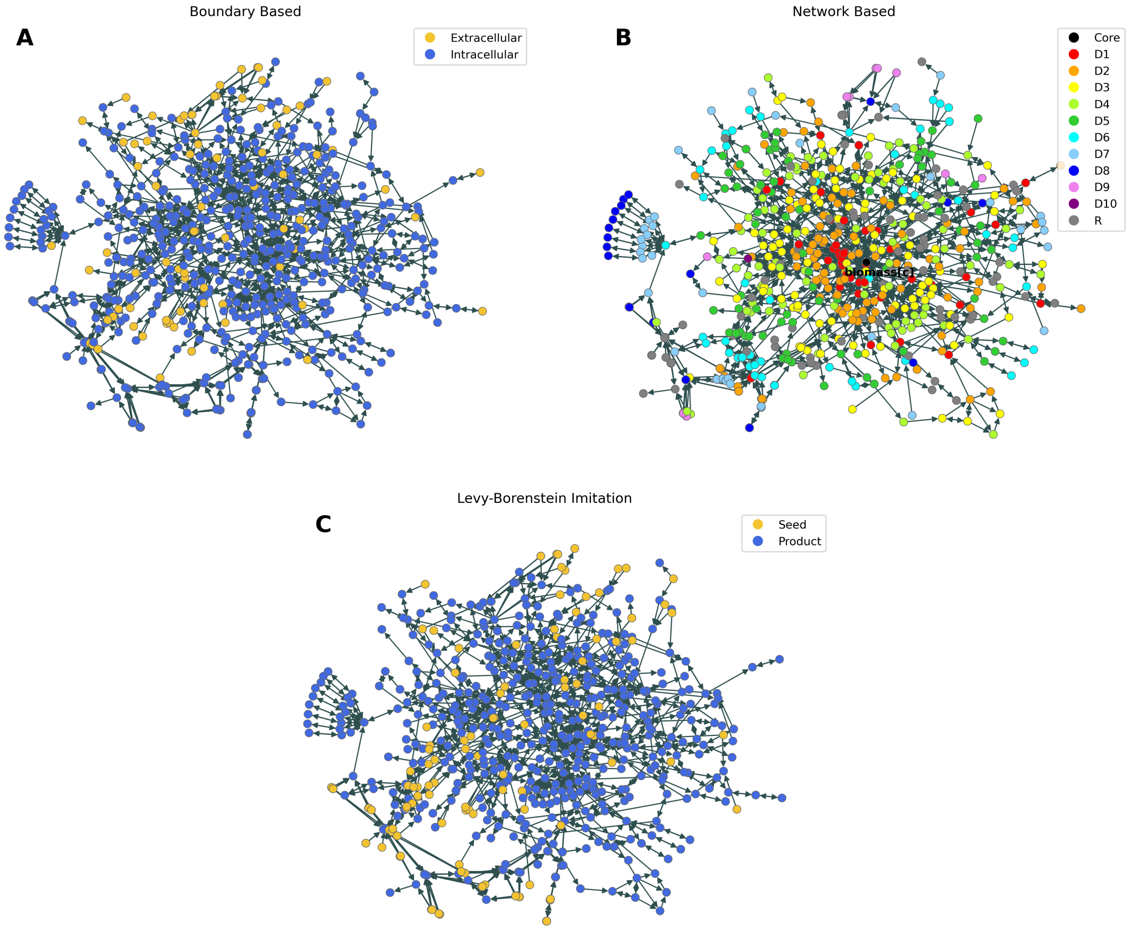

Supplementary Figure 1. Exemplary representation of the layers of a metabolic network (A) Boundary-Based, (B) Network-Based, (C) Levy-Borenstein Imitation for *Actinomyces odontolyticus ATCC 17982.* To enable a consistent comparison of the three networks in this representations, the WCC filtering step was also applied to the Boundary-Based and Network-Based networks.

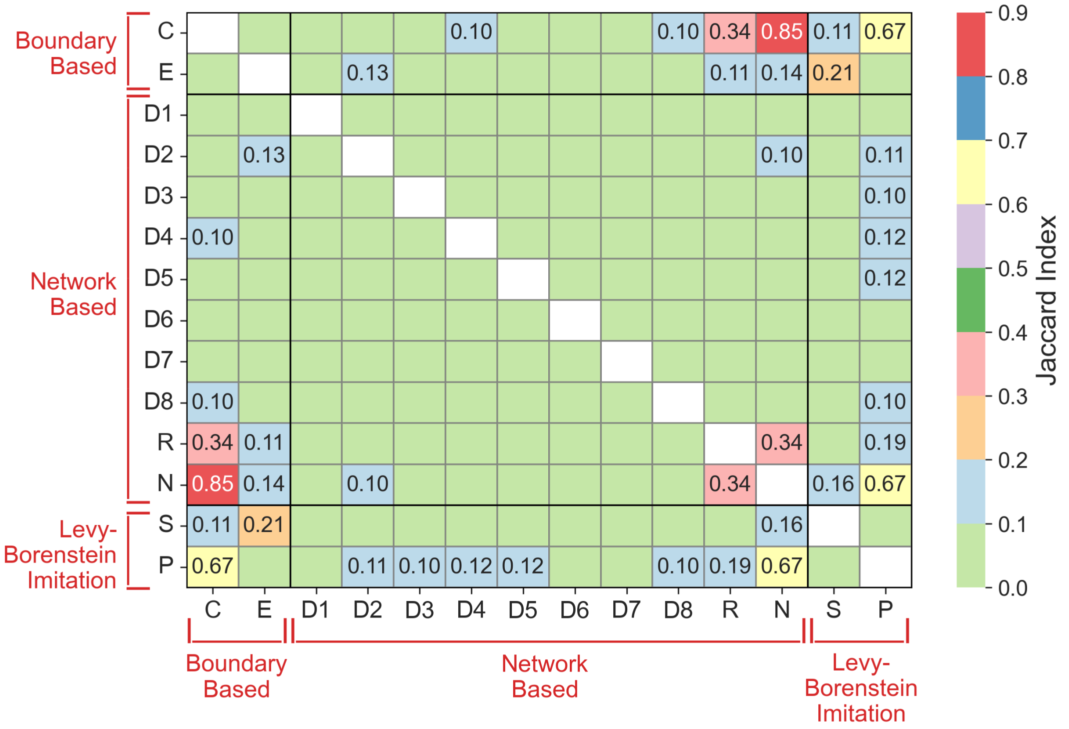

Supplementary Figure 2. Overlap of the layers/nodes for each type of index (Boundary-Based, Network-Based, Levy-Borenstein Imitation). The Overlap is assessed by computing the Jaccard Index among all the layers (nodes) for each species. The figure displays the average overlap across all species. Jaccard index values < 0.1 (green cells) are not shown in the figure.

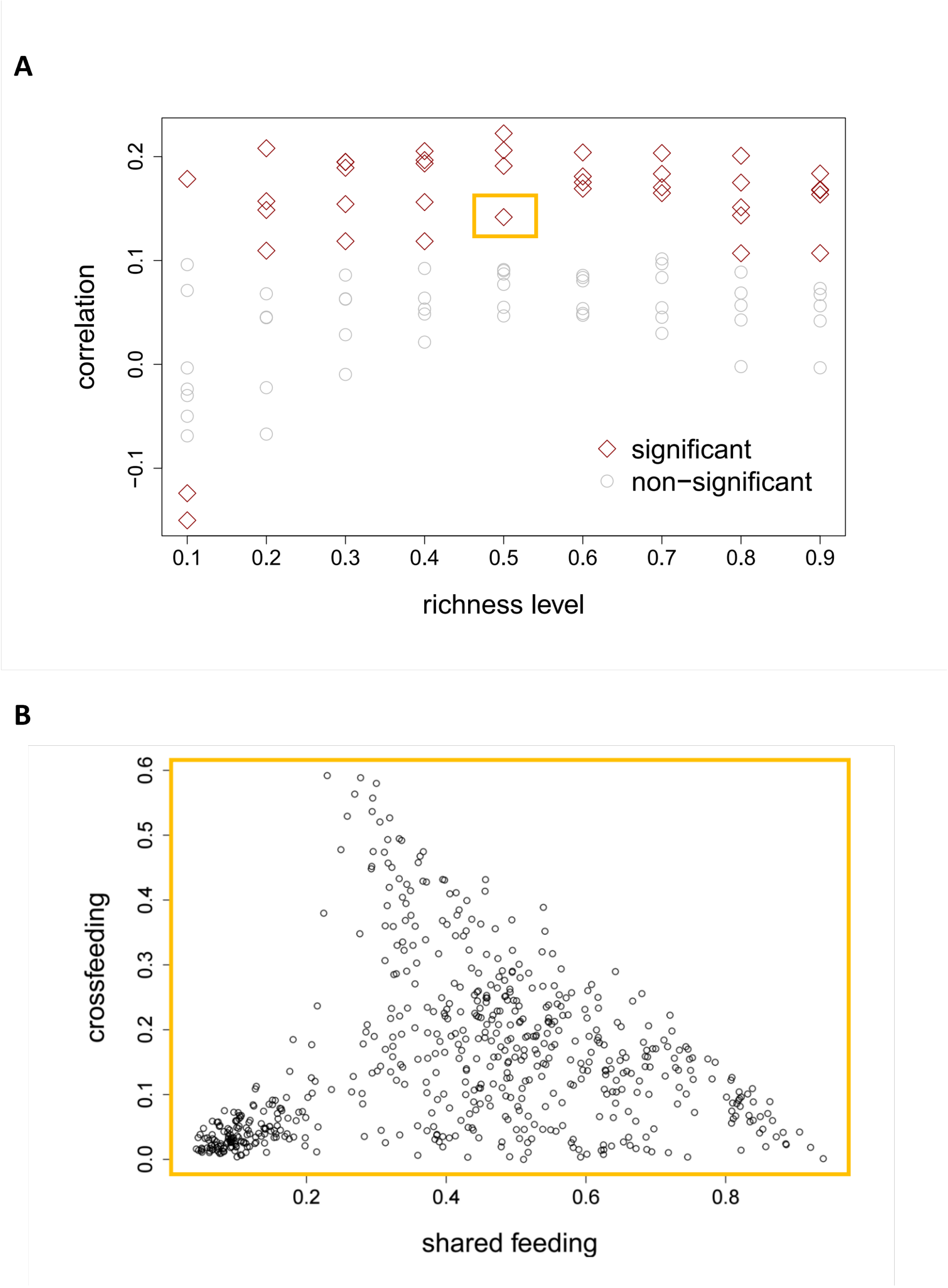

Supplementary Figure 3. Correlation between the two metabolic measures of shared feeding and crossfeeding in the BacArena simulations (A) Pearson correlations between shared- and crossfeeding for all ten random sets across the nine different richness levels. Red diamonds denote significant correlations ($p <= 0.01$), grey circles non-significant correlations. The data points underlying the framed correlation are shown in (B) Scatter plot displaying shared- and crossfeeding of set 4 at a richness-level of 0.5. The distinctive pattern of a dense, positively sloped cluster of weakly interacting pairs combined with a more widespread negatively sloped cluster of pairs, resulting in an overall slight positive correlation, is visible across most sets and richness-levels.

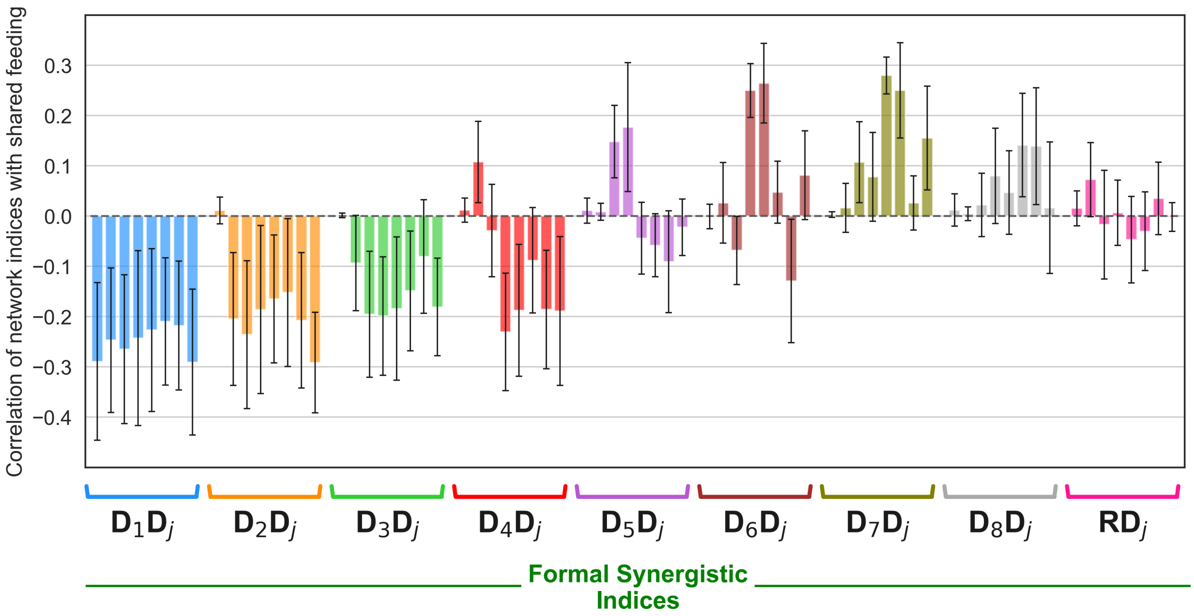

Supplementary Figure 4. Correlations between network-based formal synergistic indices and shared feeding for 73 microbial species of the human gut microbiome. Mean and standard deviation are computed across ten random sets of 25 species each (*Methods*).

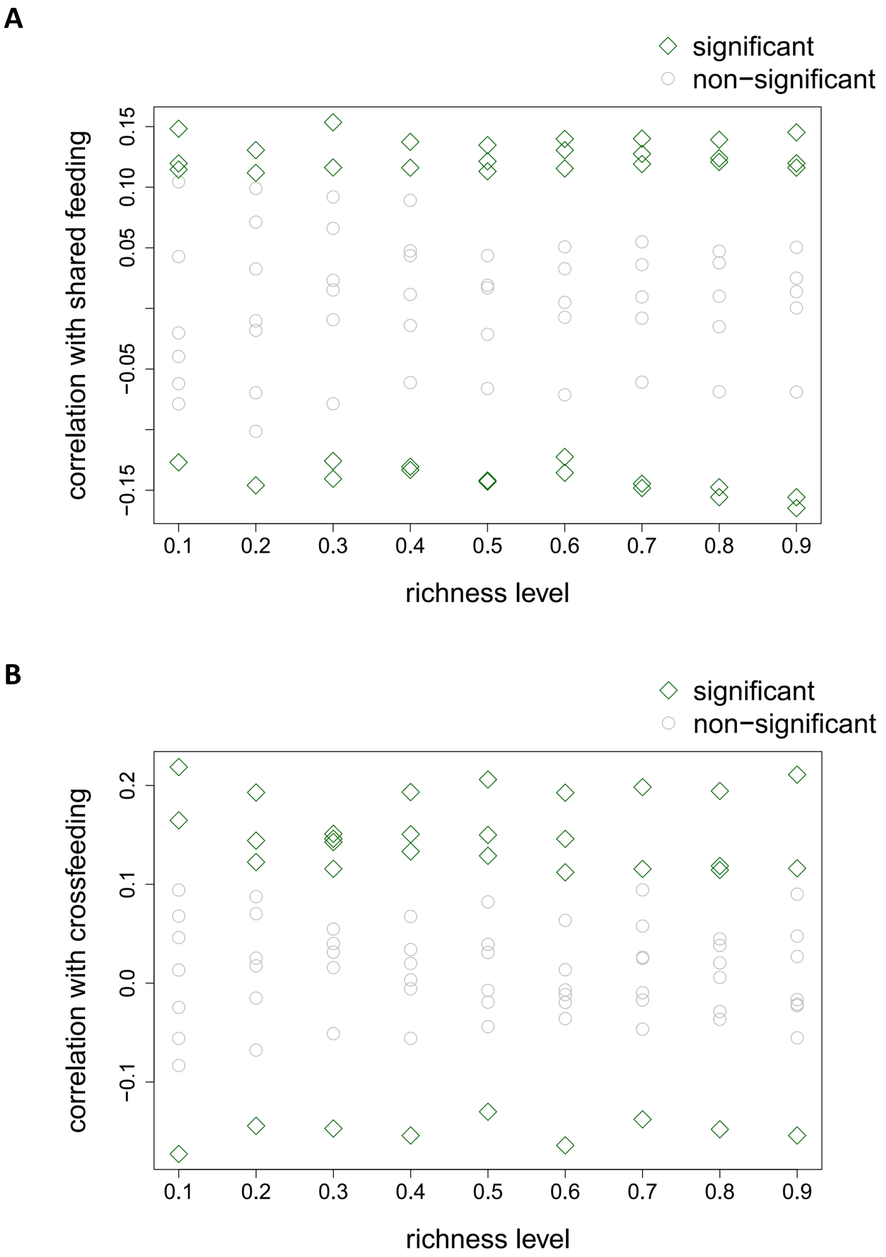

Supplementary Figure 5. Pearson correlation between co-occurrences and the simulated measures of shared feeding (A) and crossfeeding (B) for all ten random sets across the nine different richness levels. Green diamonds denote significant correlations ($p <= 0.01$), grey circles non-significant correlations. The (originally asymmetric) metabolic measures were pair-wisely averaged before correlating with the symmetric measure of co-occurrence.

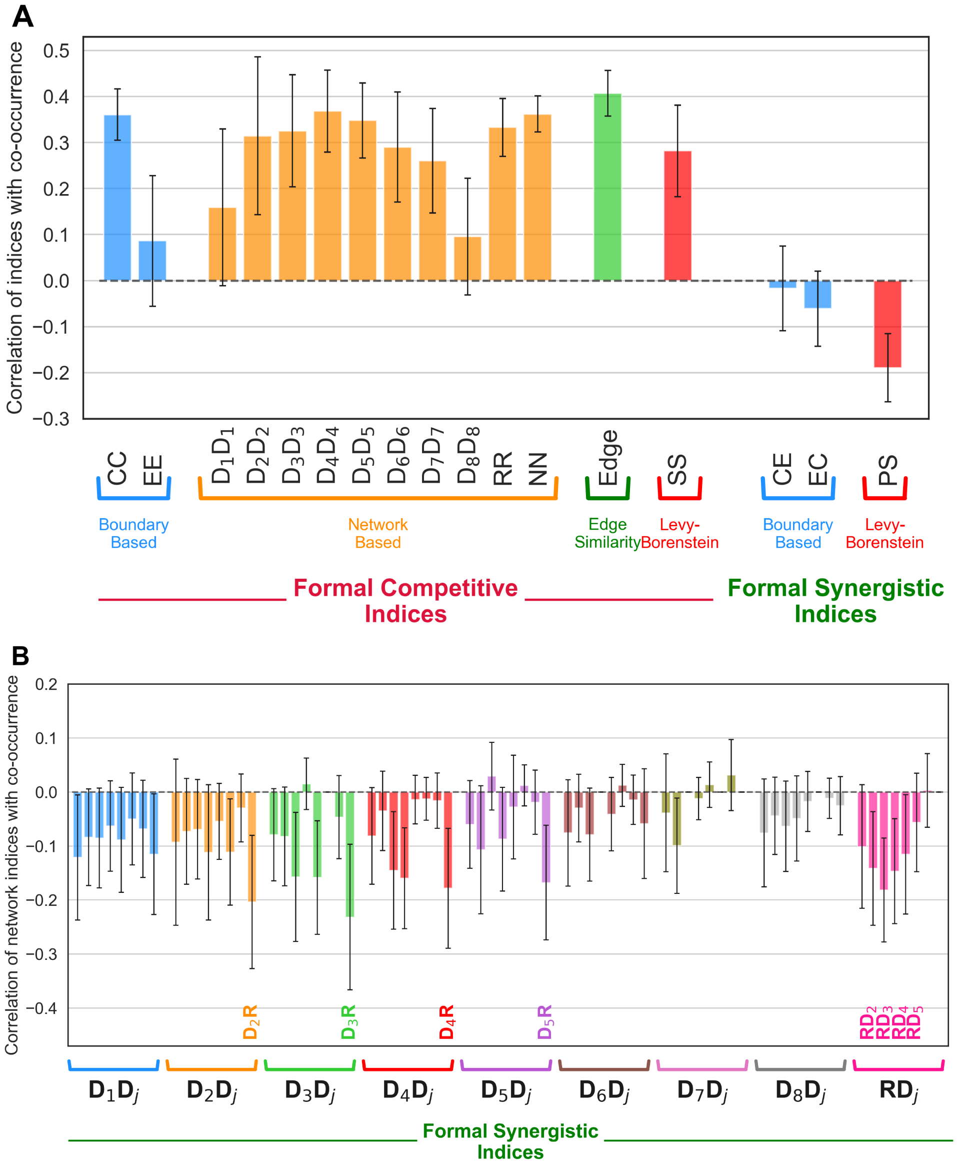

Supplementary Figure 6. Cohort: All groups; Performance of interaction indices as indicators of co-occurrence among 73 microbial species of the human gut microbiome for all the patients. Mean and standard deviation are computed across ten random sets of 25 species each (Methods). (A) Correlations between formal competitive and synergistic indices and co-occurrences. (B) Correlations between network-based formal synergistic indices and co-occurrences.

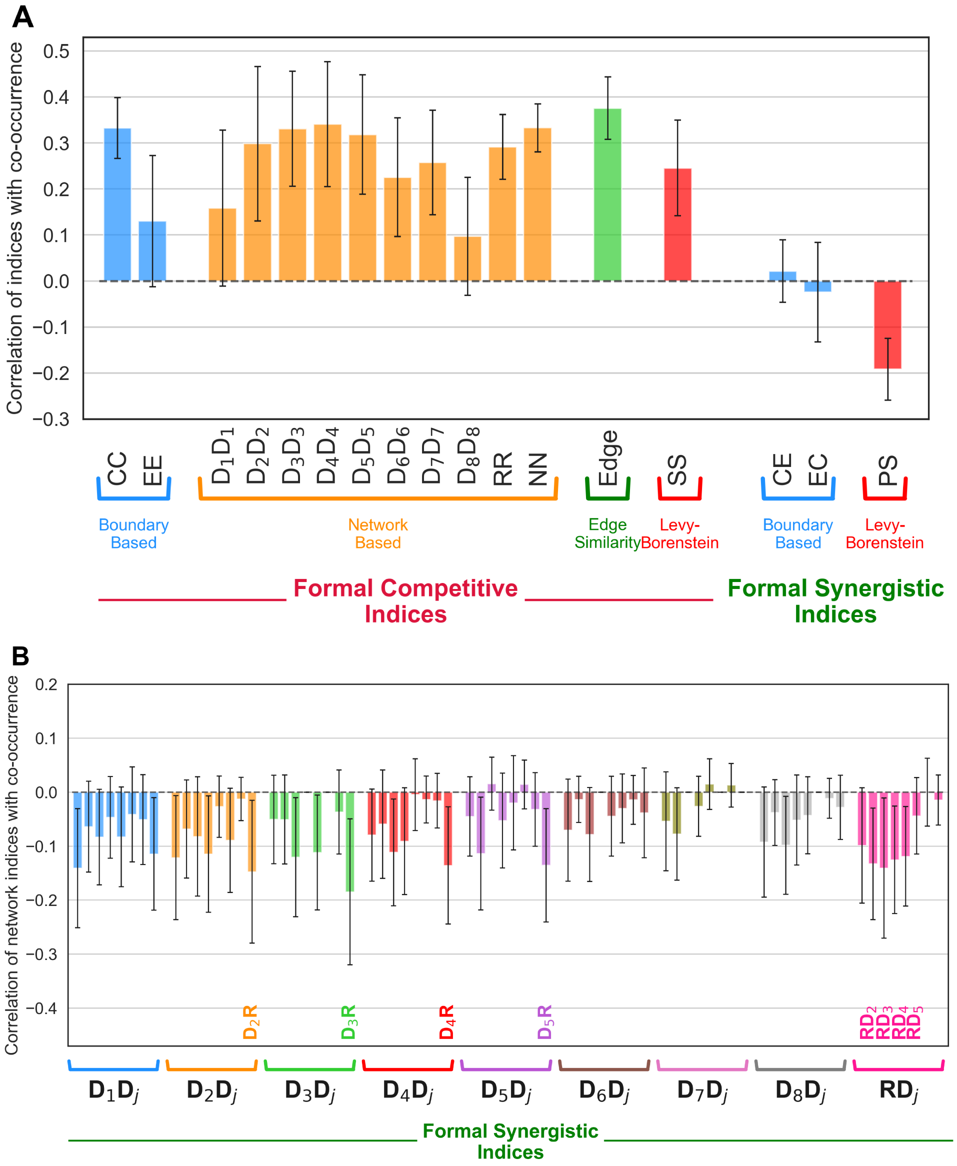

Supplementary Figure 7. Cohort: Healthy Lean; Performance of interaction indices as indicators of co-occurrence among 73 microbial species of the human gut microbiome for patients with the health status: Healthy Lean. Mean and standard deviation are computed across ten random sets of 25 species each (Methods). (A) Correlations between formal competitive and synergistic indices and co-occurrences. (B) Correlations between network-based formal synergistic indices and co-occurrences.

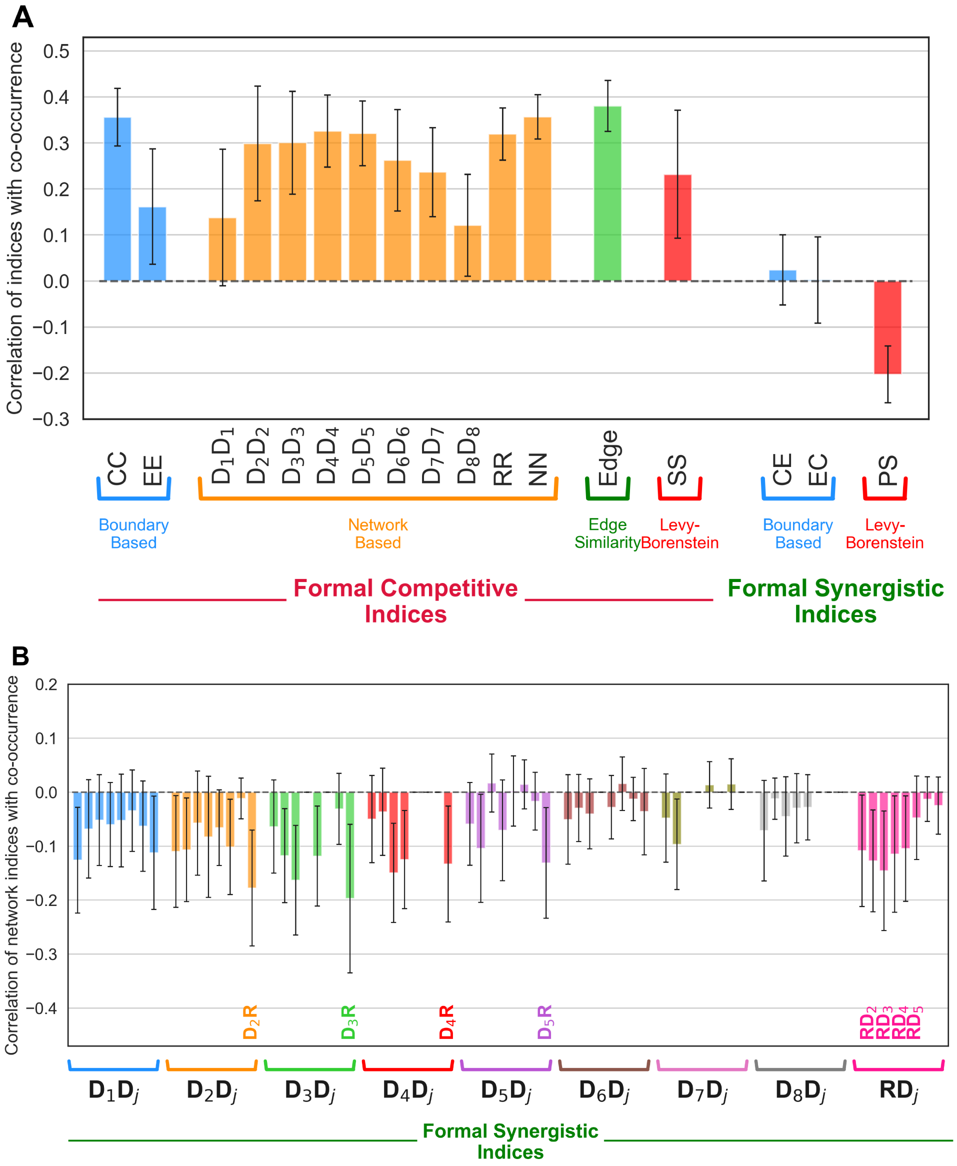

Supplementary Figure 8. Cohort: Healthy Obese; Performance of interaction indices as indicators of co-occurrence among 73 microbial species of the human gut microbiome for patients with the health status: Healthy Obese. Mean and standard deviation are computed across ten random sets of 25 species each (Methods). (A) Correlations between formal competitive and synergistic indices and co-occurrences. (B) Correlations between network-based formal synergistic indices and co-occurrences.

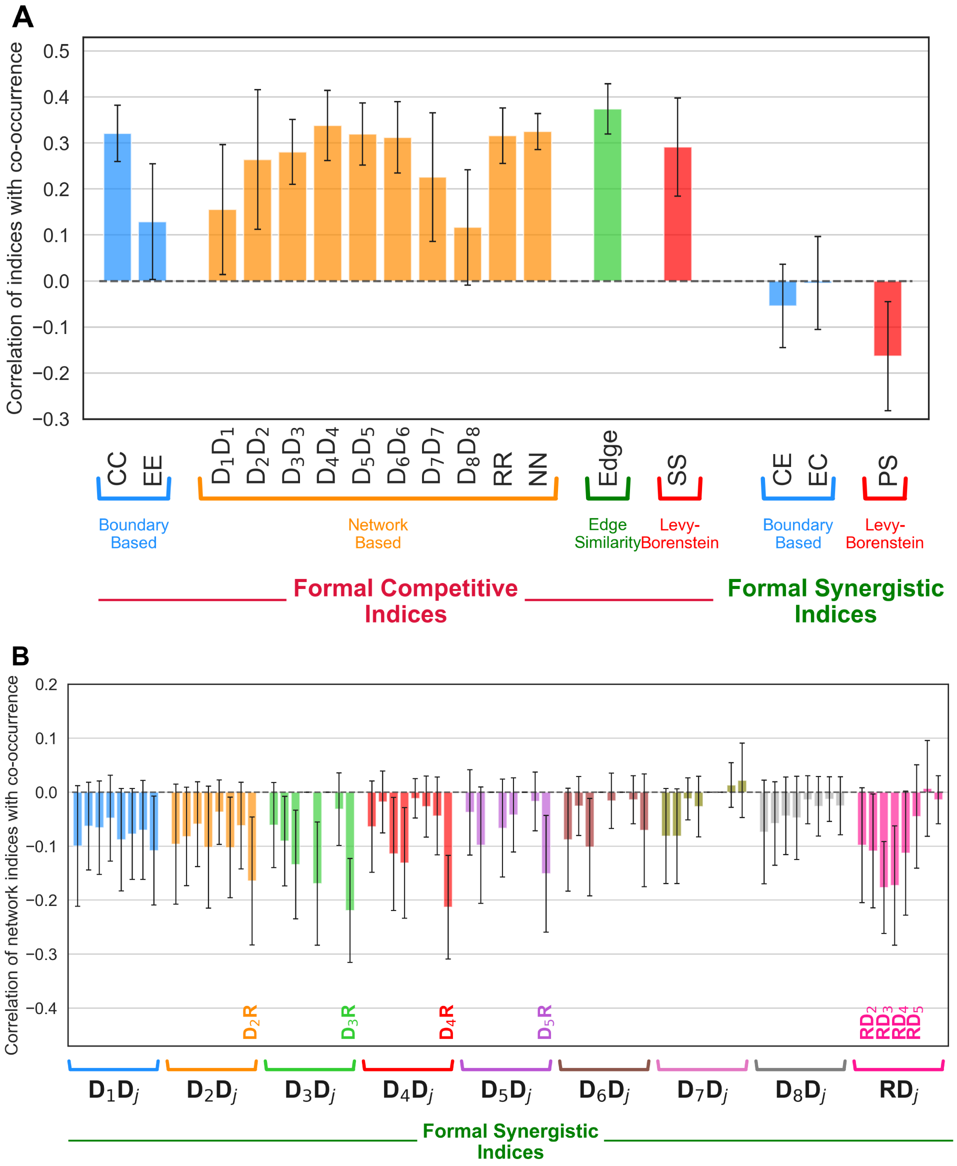

Supplementary Figure 9. Cohort: IBD Lean; Performance of interaction indices as indicators of co-occurrence among 73 microbial species of the human gut microbiome for patients with the health status: IBD Lean. Mean and standard deviation are computed across ten random sets of 25 species each (Methods). (A) Correlations between formal competitive and synergistic indices and co-occurrences. (B) Correlations between network-based formal synergistic indices and co-occurrences.

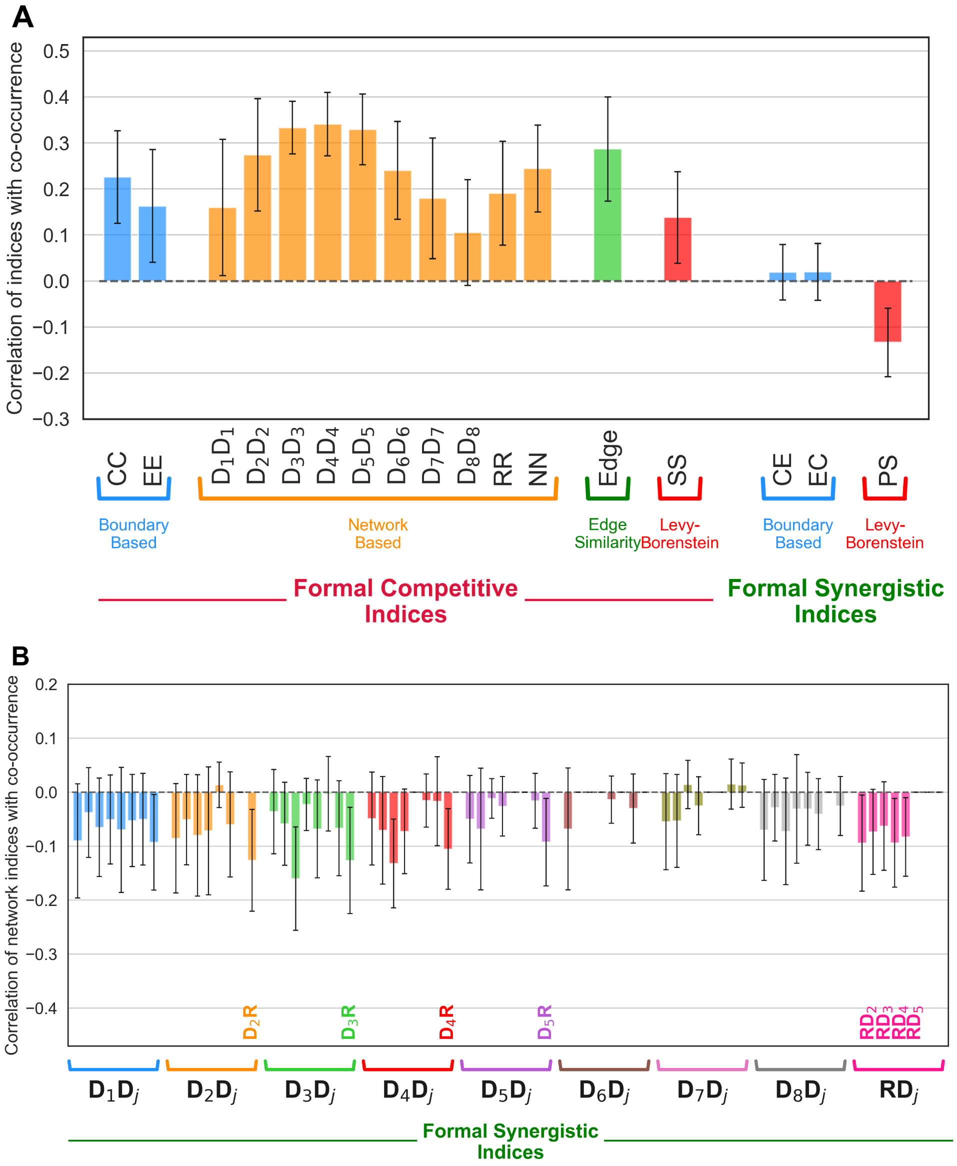

Supplementary Figure 10. Cohort: IBD Obese; Performance of interaction indices as indicators of co-occurrence among 73 microbial species of the human gut microbiome for patients with the health status: IBD Obese. Mean and standard deviation are computed across ten random sets of 25 species each (Methods). (A) Correlations between formal competitive and synergistic indices and co-occurrences. (B) Correlations between network-based formal synergistic indices and co-occurrences.

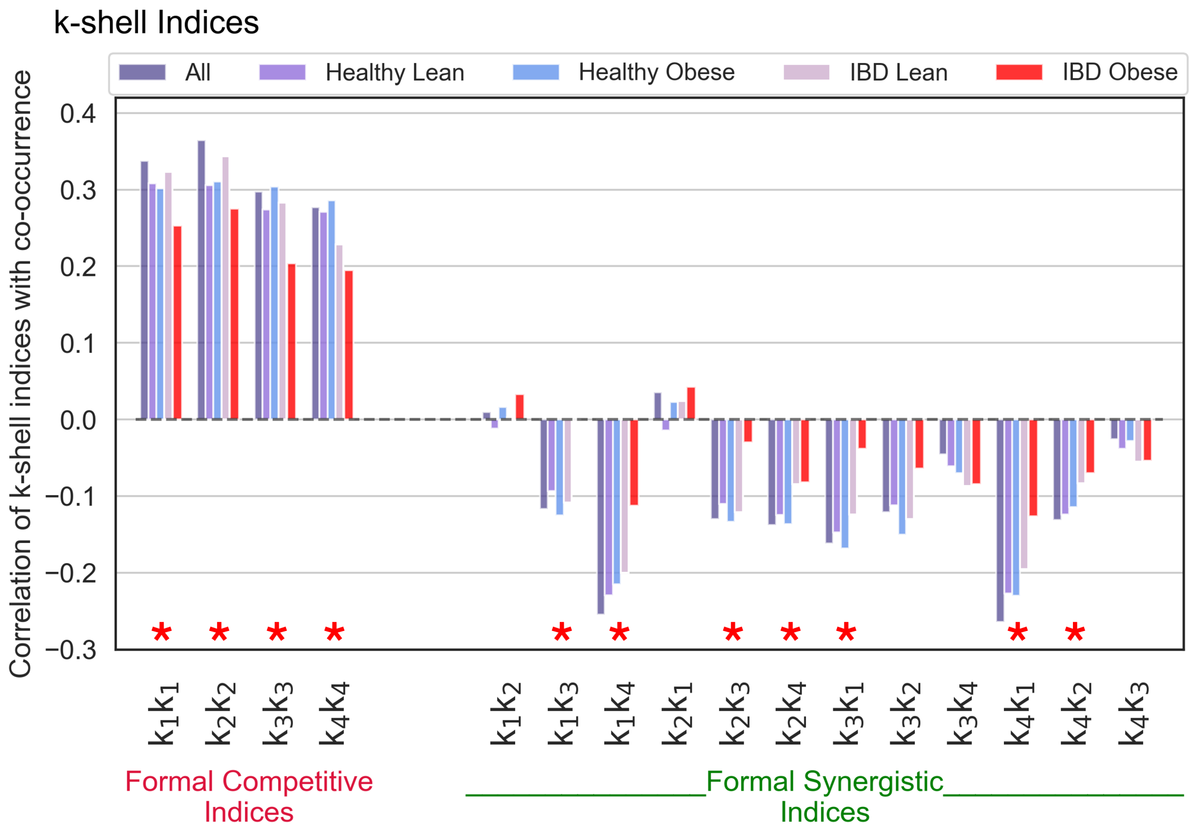

Supplementary Figure 11. Performance of k-shell indices across subgroups of patients with diverse health statuses. Correlation between co-occurrences and formal competitive and synergistic k-shell indices. Asterisk (*) associated with an index represents significant difference ($p < 0.05$) in correlations of subgroup IBD-obese compared to the subgroup containing all individuals across the ten species sets (see Supplementary Data 03 and 04 for comparison with other subgroups). The exact correlation results can be found in Supplementary Data 02.

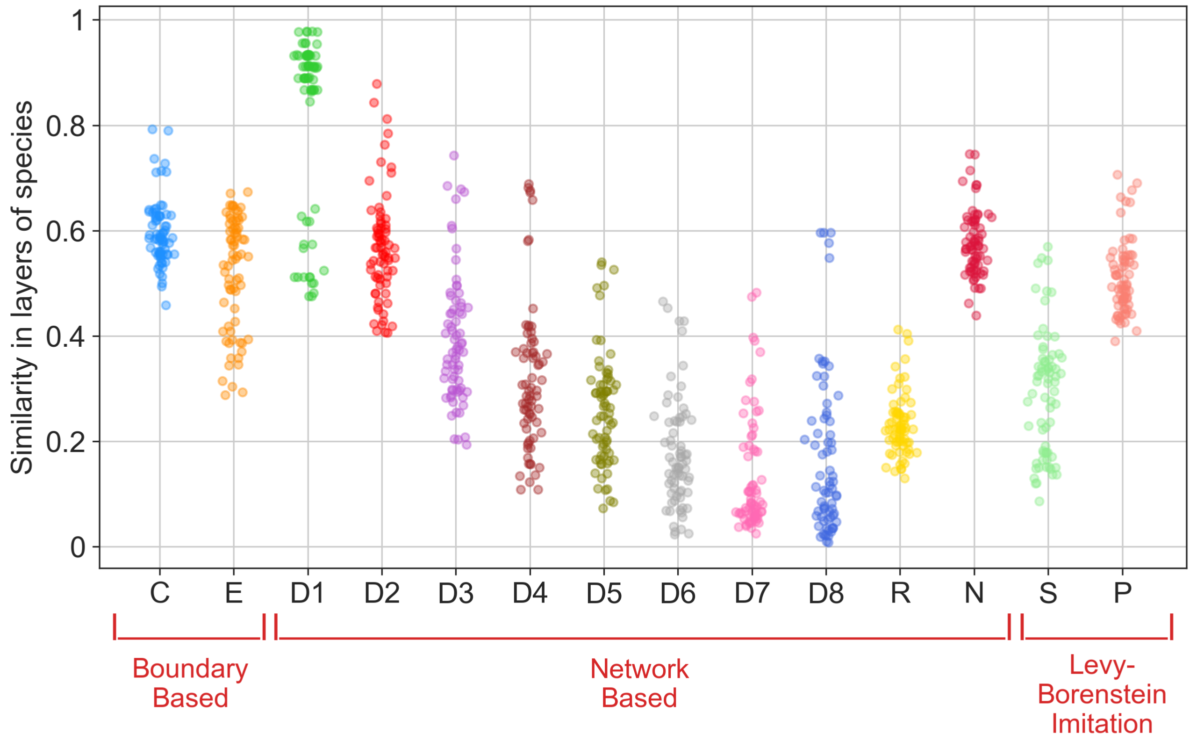

Supplementary Figure 12. Similarity in composition of each network layer across species. For each layer, we calculated the Jaccard Index of each species in comparison to a reference species, *Actinomyces odontolyticus ATCC 17982*. The x-axis represents the layer type, while the y-axis indicates the Jaccard Index.

Supplementary Data

- **Supplementary_Data_01.xlsx**. All pairwise interaction indices for 73 microbial species.
- **Supplementary_Data_02.xlsx**. Mean correlations of interaction indices with in-silico metabolic activity (Sheet 1) and microbial co-occurrence in the human gut for multiple cohorts (Sheet 2).
- **Supplementary_Data_03.xlsx**. Statistical results of comparison of correlation strengths for all competitive indices between IBD-obese and all other health groups.
- **Supplementary_Data_04.xlsx**. Statistical results of comparison of correlation strengths for selected synergistic indices between IBD-obese and all other health groups.
- **Supplementary_Data_05.xlsx**. List of the 73 microbial species and the ten random subsets being used in the study.
- **Supplementary_Data_06.xlsx**. Minimal media of the 73 microbial species being used in the study.

Supplementary References

[1] Elhanan Borenstein et al. "Large-scale reconstruction and phylogenetic analysis of metabolic environments". In: *Proceedings of the National Academy of Sciences* 105.38 (2008), pp. 14482–14487.

[2] Ali Ebrahim et al. "COBRApy: constraints-based reconstruction and analysis for python". In: *BMC systems biology* 7.1 (2013), p. 74.

[3] Minoru Kanehisa et al. "From genomics to chemical genomics: new developments in KEGG". In: *Nucleic Acids Research* 34.suppl_1 (2006), pp. D354–D357.

[4] Hong-Wu Ma and An-Ping Zeng. "The connectivity structure, giant strong component and centrality of metabolic networks". In: *Bioinformatics* 19.11 (2003), pp. 1423–1430.

[5] Stefanía Magnúsdóttir et al. *Generation of genome-scale metabolic reconstructions for 773 members of the human gut microbiota.* https://vmh.life/#downloadview. Accessed: 22-07-2024. 2017.

[6] Verónica S Martínez et al. "The topology of genome-scale metabolic reconstructions unravels independent modules and high network flexibility". In: *PLoS Computational Biology* 18.6 (2022), e1010203.

[7] Alberto Noronha et al. "The Virtual Metabolic Human database: integrating human and gut microbiome metabolism with nutrition and disease". In: *Nucleic Acids Research* 47.D1 (2019), pp. D614–D624.
